# A Chinese girl with neuromyelitis optica spectrum disorder coexisting with primary Sjogren’s syndrome: a case report and literature review

**DOI:** 10.3389/fimmu.2025.1559825

**Published:** 2025-07-08

**Authors:** Guo-qin Zhu, Rong-xuan Hu, Yan Peng, Yao Yao, Guo-min Li

**Affiliations:** ^1^ Department of Nephrology, Rheumatology and Immunology, Childrens Hospital of Jiangnan University, Wuxi, Jiangsu, China; ^2^ Department of Nephrology, Rheumatology and Immunology, Wuxi Childrens Hospital, Wuxi, Jiangsu, China

**Keywords:** aquaporin 4, children, neuromyelitis optica spectrum disorders, optic neuritis, Sjögren syndrome

## Abstract

**Introduction:**

Neuromyelitis optica spectrum disorder (NMOSD) is an immune-mediated, typically relapsing central nervous system demyelinating disorder characterized by optic neuritis (ON) and transverse myelitis (TM). While systemic or organ-specific autoimmune comorbidities are well-documented in 20–30% of adult NMOSD cases, such associations remain rarely reported in pediatric populations.

**Case Report:**

We present a 14-year-old girl with NMOSD coexisting with primary Sjögren’s syndrome (pSS). At 11 years of age, she presented with acute right-sided headache, painful eye movements, and vision loss. Diagnostic workup confirmed AQP4-IgG seropositivity, ON, and corresponding T2-hyperintense lesions on optic nerve MRI, meeting 2023 Neuromyelitis Optica Study Group (NEMOS) revised recommendations. Acute-phase treatment included intravenous methylprednisolone and intravenous immunoglobulin, followed by maintenance therapy with oral prednisone and mycophenolate mofetil (MMF), with gradual prednisolone tapering. Right-eye vision normalized after intervention. Initial workup revealed positive antinuclear antibody (ANA), anti-Ro/SSA, anti-La/SSB, and elevated alanine aminotransferase (ALT)/aspartate aminotransferase (AST). Aged 12.5 years, labial salivary gland biopsy for persistent transaminitis showed focal lymphocytic sialadenitis (focus score ≥1 focus/4 mm²), satisfying the 2016 ACR/EULAR criteria for pSS. At 13.5 years, MMF was switched to tacrolimus due to persistent ALT/AST elevation, leading to biochemical normalization. No NMOSD relapses occurred post-initial episode.

**Conclusion:**

This case highlights the rare but clinically important co-occurrence of NMOSD and pSS in children. Routine screening for autoantibodies (e.g., ANA, organ-specific antibodies) in pediatric NMOSD is warranted to detect comorbid autoimmune disorders. Targeted therapy for concurrent connective tissue diseases can optimize clinical outcomes and quality of life.

## Introduction

Neuromyelitis optica spectrum disorder (NMOSD) is a rare and severe inflammatory autoimmune disease of the central nervous system (CNS) that is associated with serum aquaporin-4 (AQP4-IgG) antibodies directed against the AQP4 channel found on the foot processes of astrocytes (1–3). AQP4-IgG is key in NMOSD pathophysiology and causes astrocytopathy, demyelination, and neuropathy through complement activation and cell-mediated immunity (4–6). NMOSD has six core clinical characteristics: optic neuritis (ON), acute myelitis, area postrema syndrome, acute brainstem syndrome, acute diencephalic clinical syndrome and symptomatic cerebral syndrome. Among these, three main clinical features are recognized: isolated longitudinally extensive TM or isolated ON; various forms of brainstem encephalitis found in adults; and a broad variety of cerebral symptoms mostly found in children (7–9). Presentations are uncommon in the pediatric age range, accounting for approximately 3%-5% of cases (10).

Connective tissue disease (CTD) is a broad category of rheumatic diseases related to autoimmune dysfunction that can cause damage to multiple organs and systems in the body, including the nervous system. Although demyelination of the central nervous system can be caused by CTD alone, an increasing number of reports have revealed that NMOSD often coexists with CTDs in adults, particularly primary Sjögren syndrome (SS) and systemic lupus erythematosus (SLE) (11–13). An analysis of studies that recruited unselected patients with SLE and SS revealed pooled overlapping percentages of NMOSD of 0.6% and 6.5%, respectively (9). Studies enrolling rheumatologic patients with nervous system symptom involvement reported a higher percentage of NMOSD, with a pooled percentage of 26.5% in SS patients (9). Similarly, when patients with NMOSD were recruited, the pooled percentages of SS or SLE patients were 7.0% and 3.5%, respectively (9). Although the coexistence of NMOSD and pSS is relatively common in adults, this condition is rarely observed in children. Here, we report a child with NMOSD who also has coexisting pSS.

## Case report

**Figure 1:**
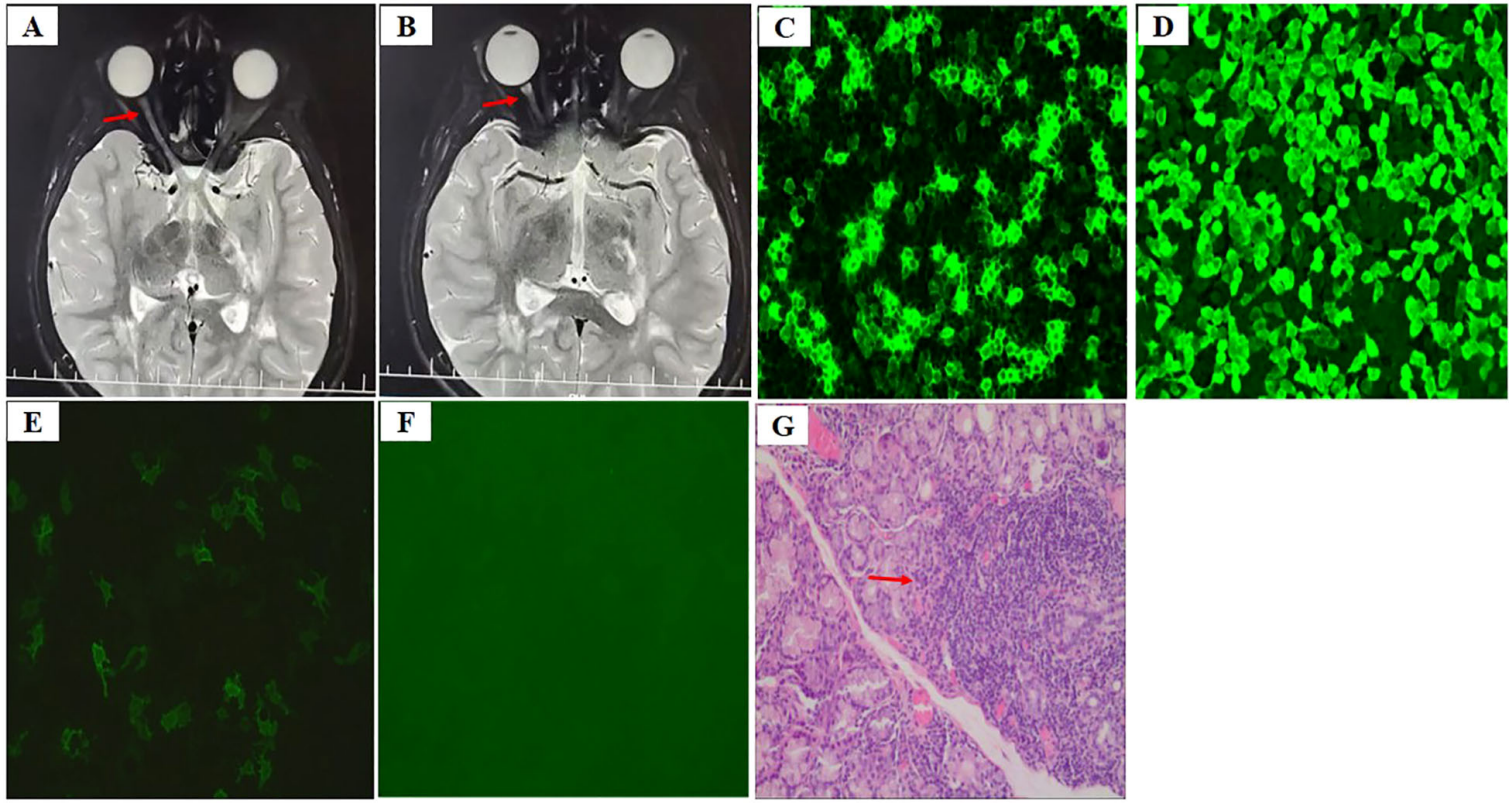
**(A, B)** Axial T2-weighted image shows thickening of the right optic nerve **(A)** and hyperintense lesion in the optic nerve sheath **(B)** [arrows in **(A, B)**], **(C)** AQP4-IgG in the serum at an AQP4 titer of 1:1000 by cell-based assay (CBA) before therapy, **(D)** AQP4-IgG for positive control, **(E)** AQP4-IgG in the serum at an AQP4 titer of 1:100 during preventive therapy, **(F)** AQP4-IgG for negative control, **(G)** Labial salivary gland biopsy displays focal lymphocytic sialadenitis with 1 focus/4 mm2 [arrow in **(G)**].

The patient, a 14-year-old Chinese girl, presented with acute onset of right-sided headaches, pain with eye movements, and vision loss in February 2021 (11 years old). She was admitted to the ophthalmic department of the local hospital. Head and orbital MRI revealed high T2 signals with enhancement in the inner segment of the right optic nerve frame and multiple abnormal signals in the left basal ganglia area and subcortical regions of both the frontal and parietal lobes (**Figures 1A, B**). Neuro-ophthalmology examination revealed visual acuity of 20/40 in the right eye (oculus dexter, OD), with a right relative afferent pupillary defect (RAPD) and 20/30 in the left eye (oculus sinister, OS). The color vision (Hardy–Rand–Rittler) was 4/14 OD and5/14 OS. Intraocular pressures were within normal limits in both eyes. Slit-lamp examination was unremarkable. Extraocular motility was full, but there was left-eye discomfort with eye movements. A dilated fundus exam revealed circumferential optic disc edema with peripapillary hemorrhage OD and a normal disc in the OS.

The serological workup for tuberculosis, syphilis, and myelin oligodendrocyte glycoprotein (MOG) was negative. The anti-aquaporin-4 immunoglobulin G (AQP4-IgG) was detected in the serum at an AQP4 titer of 1:1000 (**Figures 1C, D**). Cerebrospinal fluid (CSF) analysis was normal. CSF culture was negative, as were all polymerase chain reaction tests for fungal and viral infections. The erythrocyte sedimentation rate (ESR) and C-reactive protein (CRP) level were normal before the initiation of steroids. ANA was positive at a titer of 1:320 in a speckled pattern, and anti-Ro/SSA and anti-La/SSB were positive. Others antibodies, including the remainder of the extractable nuclear antigen (ENA) panel, anti-double-stranded DNA antibodies (anti-ds-DNA), anti-neutrophil cytoplasmic antibody (ANCA), antiphospholipid (aPL) antibodies, and rheumatoid factor (RF), were negative. Complement 3 and 4 levels were normal. Urinalysis revealed no abnormalities. Quantitative immunoglobulins were within normal limits.

She was diagnosed with NMOSD on the basis of a positive test for AQP-IgG and acute optic neuritis and was treated with intravenous pulse methylprednisolone (IVMP) at 500 mg per day for 3 consecutive days. She continued to be administered IVMP at 250 mg per day for 3 consecutive days combined with intravenous immunoglobulins (IVIGs) at 400 mg/kg per day for 5 consecutive days because of a poor response to the first dose of IVMP. Her vision gradually returned to normal after acute treatment. Oral prednisone and mycophenolate mofetil (MMF; 1000 mg) were administered for preventive treatment after acute treatment, and the dose of prednisolone was gradually tapered. She was admitted to our hospital for evaluation because of persistently elevated alanine aminotransferase (ALT) and aspartate aminotransferase (AST) levels in June 2022. Physical examination revealed Cushing’s appearance. Other physical findings were unremarkable. Schirmer test results was normal (>5 mm/5 min) and ocular surface staining was negative. Her medical history and family history were also unremarkable. Laboratory testing revealed elevated ALT (272 U/l, normal range 7–40 U/l) and AST (1–42 U/l, normal range 13–35 U/l) levels. Serum bilirubin levels were normal. The AQP4- IgG was tested in the serum at an AQP4 titer of 1:100 (**Figures 1E, F**). Autoantibodies were positive for ANA (1:640), anti-Ro/SSA and anti-La/SSB, while tests for other autoantibodies, including the remainder of the ENA panel, anti-ds-DNA, ANCA, aPL, RF, lupus anticoagulant (LAC), anti-β2 glycoprotein-I antibody (anti-β2GPI) and autoimmune hepatitis antibodies, were negative. Labial salivary gland biopsy was performed to identify possible reasons for the elevated ALT and AST levels, and focal lymphocytic sialadenitis with 1 focus/4 mm^2^ (1 focus ≥50 lymphocytes/4 mm²) was detected (**Figure 1G**). The patient also met the 2016 American College of Rheumatology/European League Against Rheumatism (ACR/EULAR) classification criteria for primary Sjögren’s syndrome on the basis of positive anti-SSA and labial salivary glands with focal lymphocytic sialadenitis and a focus score of ≥1 foci/4 mm^2^ (14). MMF was switched to tacrolimus because of persistently elevated ALT and AST levels at the age of 13.5 years. ALT and AST levels subsequently returned to normal. NMOSD did not occur after the first episode.

## Discussion

Herein, we report a Chinese girl with NMOSD coexisting with SS. She was 11 years old, had unilateral vision loss (right) and pain with eye movements, and presented with ON. She fulfilled both the 2015 International Panel for NMO Diagnosis (IPND) criteria and the 2023 revised recommendations by the Neuromyelitis Optica Study Group (NEMOS), demonstrating AQP4-IgG seropositivity along with optic neuritis and corresponding T2-hyperintense lesions on optic nerve MRI (8, 15).

The incidence of NMOSD in China per 100,000 person-years was 0.278, with 0.075 in children and 0.347 in adults (16). Therefore, NMOSD is a rare disease in adults and is more rare in children. The onset of the disorder typically occurs at a median age of 40 years in adults, whereas the typical age at onset is between 10 and 12 years in pediatric patients with NMOSD (1, 10, 17, 18).

NMOSD can be divided into three groups according to the presence of serum antibodies: anti–AQP4-IgG antibodies, anti-myelin oligodendrocyte glycoprotein (MOG-IgG) antibodies, and seronegative NMOSD (7, 19). Similar to other autoimmune diseases, seropositive NMOSD is more common in females than in males, with a female-to-male ratio of nearly 10:1 in adults, whereas seronegative cases exhibit an equal sex distribution (17, 20). Serum anti-AQP4 antibodies are present in 70–90% of patients with adult NMOSD and are associated with high specificity (21). Approximately 31% of pediatric patients with NMOSD have detectable serum anti–AQP4-IgG, 57% have serum MOG-IgG, and approximately 12% are double seronegative (22, 23). Therefore, the frequency of AQP4-IgG antibody seropositivity in patients with NMOSD is generally lower in children than in adults (24).

The most frequent clinical features of NMOSD are visual, motor, sensory and constitutional symptoms, such as fever, vomiting, and seizures (1, 25). ON occurs as the first clinical symptom in 50–75% of pediatric patients, and TM occurs in 30–50%, either alone or in combination (3, 25, 26). Other manifestations included ataxia, encephalopathy, and cranial nerve dysfunction, such as ophthalmoparesis or area postrema syndrome (2, 3).

A recurrent course of NMOSD, with exacerbations and incomplete remissions, leads to the development of disability, which has a profound impact on patients’ quality of life. However, recently, a number of randomized controlled trials have demonstrated that biological therapies that act on key elements of NMOSD pathogenesis, such as the interleukin-6 pathway, B cells, and complement, have impressive efficacy in preventing the occurrence of clinical relapses (6, 27). Therefore, four preventive immunotherapies have now been approved for AQP4-IgG-positive NMOSD in many regions of the world: eculizumab, ravulizumab, inebilizumab, and satralizumab. These new drugs may potentially substitute rituximab and classical immunosuppressive therapies, which were as yet the mainstay of treatment for both, AQP4-IgG-positive and -negative NMOSD (23). Early diagnosis and aggressive treatment are crucial for managing symptoms and preventing long-term disability in NMOSD patients.

In addition to AQP4-IgG antibodies, antinuclear autoantibody (ANA) and other non-organ-specific autoantibodies are also often detectable in patients with NMOSD who do not have clinical evidence of a systemic autoimmune disease (28, 29). Although some vaccinations, neoplasms, infections, medications or systemic autoimmune diseases can mimic manifestations of NMOSD (16, 30–32), the coexistence of NMOSD with certain systemic diseases, such as myasthenia gravis, autoimmune hypothyroidism, SLE or pSS, is currently well established (11, 33).

At the beginning of the disease, ANA, anti-Ro/SSA and anti-La/SSB autoantibodies were detected in our patient. She had no clinical features involving the exocrine glands. However, persistently elevated ALT and AST levels were observed in the patient and unexplainable by NMOSD. In addition, specific autoimmune hepatitis markers (such as anti-LKM and anti-SMA antibodies) were negative, ruling out autoimmune hepatitis as the cause of elevated ALT and AST levels. The ALT and AST levels were already elevated prior to MMF treatment and did not further increase after its initiation, making drug-induced liver injury unlikely. Labial salivary gland biopsy was performed, revealing focal lymphocytic sialadenitis with a focus score ≥1 focus/4 mm^2^. The patient also met the 2016 ACR/EULAR classification criteria for primary Sjögren’s syndrome on the basis of positive anti-SSA and labial salivary glands with focal lymphocytic sialadenitis and a focus score of ≥1 foci/4 mm^2^ (14).

pSS is rare in children and has a different clinical presentation than in adults (34–37). Adult pSS is particularly prominent with glandular symptoms (such as dry mouth and dry eyes), whereas pediatric pSS is more commonly associated with constitutional symptoms (fever, rash or purpura) and extraglandular manifestations, such as hematological, hepatic, articular and renal involvement (34–37).

The prevalence of neurological manifestations in primary SS varies between 10% and 70%, of which 2–25% of adults exhibit central nervous system (CNS) involvement (37). A wide spectrum of neurologic manifestations in pSS can range from asymptomatic brain lesions on MRI to symptomatic brain lesions, meningitis, myelopathy, cranial neuropathy, sensorimotor polyneuropathy and mononeuritis multiplex (37, 38). Neurological involvement is not common in pediatric patients with pSS, and CNS symptoms are present in 8.7% of patients (39). Headache is the most common symptom of CNS involvement, which is among the rarest systemic involvements in pediatric pSS patients (37, 39).

Our patient met the diagnosis of the coexistence of NMOSD and pSS. Studies in adults revealed that NMOSD is most frequently associated with SS and anti-Ro/SSA antibody (SSA-Ab) positivity in Chinese populations (40, 41). The prevalence of SS is greater among AQP4-IgG-positive patients than among AQP4-IgG-negative patients, with a potential prevalence of 10–20% at the time of diagnosis of AQP4-IgG-positive NMOSD (41). Prasad CB et al. performed a systematic review and found that NMOSD preceded primary SS onset in 45.5% of patients, that NMOSD occurred after primary SS onset in 29.5% of patients, and that 25% of patients presented simultaneously (42).

The reported prevalence of SS in adult patients with NMOSD is estimated to be between 2% and 30%, representing is a highly underrecognized association (42). Although the coexistence of NMOSD and SS is not uncommon in adults, this condition is very rare in children.

Five patients (including our patient) with both NMOSD and pSS were identified via a literature review of PubMed (**Table 1**) (43–46). They were all female. NMOSD preceded pSS onset in one patient and occurred after pSS onset in one patient. The diagnosis of NMOSD preceded the diagnosis of pSS in our patient, but the symptoms of the two diseases occurred simultaneously (43–46). Three patients presented simultaneously. The median age of NMOSD onset was 11.4 years (range 9-17), and the median age of pSS onset was 12.8 years (range 9-17). Among these five patients, two had autoimmune diseases other than pSS and NMOSD. One patient had SLE, and the other had multiple autoimmunity disorders, including Hashimoto’s disease, celiac disease, and vitiligo (45, 46). Four of the five patients had ON, and three had TM. pSS symptoms differed across the five patients. One patient had parotid gland enlargement, multiple oral cavity caries and fever. In addition, one patient had distal renal Tubular acidosis (dRTA), and one patient had elevated ALT and AST levels. The other two patients had no symptoms and were diagnosed by salivary gland biopsy (43–46). Four of the five patients achieved complete remission after acute and preventive therapy, and one achieved no remission, with a recurrent course and the accumulation of disability (43–46).

**Table 1:** Clinical feature of NMOSD coexisting with SS in children.

P	A1	G	Clinical features of SS	Positive anti-bodies	Salivary gland biopsy	Therapy for SS		A2	Clinical features of NMOSD				Therapy for NMOSD		
						Induction therapy	Maintenance therapy		Phenotype	Spine MRI	Brain MRI	Positive anti-AQP4	Acute therapy	Preventive therapy	Outcome
1@	12.5	F	elevated ALT and AST	ANA Anti-SSA Anti-SSB	Positive	Prednisone MMF HCQ	Prednisone MMF HCQ	11	ON	Normal	The right optic nerve indicative of an inflammatory process	Y(1:3200) in serum	IVMP IVIG MMF	Prednisone MMF	CR
2 (42)	11	F	no symptoms	ANA Anti-SSA Anti-SSB	Positive	Prednisone	Prednisone	11	ON	Diffuse patchy T2-weighted signal from C1 to the conus.	the optic nerves indicative of an inflammatory process	NA	IVMP TPE CTX	Prednisone	CR
3 (43)	20	F	no symptoms	ANA Anti-SSA Anti-SSB	Positive	Prednisone AZA	Prednisone AZA	10	ON	hyperintense lesion extending from T5 to T10 level. Multiple small hyperintense lesions and cord atrophy between T2 and T5 segments.	The optic nerve indicative of an inflammatory process	NA	IVMP AZA CTX	Prednisone AZA	NR
4* (44)	17	F	dRTA	ANA Anti-SSA Anti-SSB	NA	Prednisone	Prednisone	17	brain involvement	NA	Diffuse increase of signal intensity and edema in the pons	NA	Prednisone	Prednisone	CR
5# (45)	5	F	Parotid gland enlargement, oral cavity multiple caries, epistaxis, fever	ANA Anti-SSA Anti-SSB a-CL RF	NA	Prednisone MMF HCQ IVIG Rituximab	Prednisone HCQ MMF Tacrolimus	9	ON	slight swelling of the spinal cord at the levels of c2-6	The left optic nerve indicative of an inflammatory process	Y(1:3200 in serum, 1:100 in CSF)	IVMP Rituximab IVIG	Rituximab MMF Tacrolimus	CR

A1, Age at SS diagnosis (y); A2, Age at NMOSD diagnosis (y); a-CL, anticardiolipin antibodies; ALT, alanine aminotransferase; ANA, antinuclear antibodies; AQP4, aquaporin 4; AST, aspartate aminotransferase; AZA, azathioprine; B, brain; BS, brainstem; CR, complete remission; CS, corticosteroids; CTX, cyclophosphamide; dRTA, distal renal Tubular acidosis; dsDNA, double-stranded DNA; ENA, extractable nuclear antigens; G, gender; HCQ, hydroxychloroquine; LETM, longitudinally extensive transverse myelitis; IVMP, intravenous pulse methylprednisolone; MAS, macrophage activation syndrome; MMF, mycophenolate mofetil; NA, not available; NMOAS, neuromyelitis optica spectrum disorder; ON, optic neuritis; NR, No remission; P, patient; PR, partial remission; SS, Sjögren’s syndrome; TM, transverse myelitis; TPE, therapeutic plasma exchange.

@: Our patient.

*: The patient had polyautoimmunity formed by Hashimoto’s disease, celiac disease, vitiligo, and Sjögren’s syndrome.

#: The patient had Systemic lupus erythematosus and Sjögren’s syndrome.

The international panel of NMOSD diagnoses concluded that the presence of CTD in NMOSD patients is a coexistence, rather than a complication, of connective tissue disease. NMOSD patients with anti-SSA/Ro antibodies have a significantly greater risk of relapse (47), and anti-SSA/Ro antibodies may be associated with disease activity and severe disability in patients with NMOSD (48). When first-attack NMOSD patients are complicated with CTD, they have a higher recurrence rate, more recurrences, and earlier first recurrence (49). The authors agreed that autoantibodies, ANA and other organ-specific or non-organ-specific autoantibodies should be detected in patients with NMOSD to identify systemic or organ-specific autoimmune diseases and to help predict the prognosis of patients with NMOSD. The coexistence of NMOSD and pSS can present complex challenges in terms of management and treatment. Diagnosing NMOSD in pSS patients is crucial, as it has a highly relapsing course requiring indefinite immunosuppression. Moreover, if not diagnosed early, damage accrual occurs over time, leading to permanent disability and morbidity. CNS symptoms may occur during the course of the disease in children with pSS, and AQP4-IgG should be tested as early as possible to avoid missed diagnoses. Once CTDs coexisting with NMOSD are diagnosed, targeted treatment should be actively administered to improve the prognosis and quality of life of patients.

## Conclusion

NMOSD is an autoimmune condition that commonly co-occurs with systemic or organ-specific autoimmune diseases in adult populations, though such associations are infrequently observed in pediatric cases. Comprehensive autoantibody screening, including ANA and organ-specific/non-organ-specific autoantibodies, is essential in NMOSD patients to identify concurrent autoimmune disorders and facilitate prognostic evaluation. Clinicians should maintain a high index of suspicion for autoimmune comorbidities during long-term follow-up. Importantly, AQP4-IgG testing should be performed in patients with underlying autoimmune diseases who develop neurological symptoms suggestive of NMOSD. Accurate diagnosis of NMOSD is particularly crucial for the effective management of patients with CTD.

## Data Availability

The original contributions presented in the study are included in the article/supplementary material. Further inquiries can be directed to the corresponding author.
